# Integrative analysis of homologous recombination repair patterns unveils prognostic signatures and immunotherapeutic insights in breast cancer

**DOI:** 10.1007/s13353-024-00848-1

**Published:** 2024-03-13

**Authors:** Yan-Shuang Li, Hong-Chuan Jiang

**Affiliations:** Department of Breast Surgery, Beijing Chaoyang Hospital, Capital Medical University, Beijing, 100020 China

**Keywords:** Breast cancer, Homologous recombination repair, Immunity, Prognosis, Immunotherapy response

## Abstract

Globally, breast cancer (BC) is the leading cause of female death and morbidity. Homologous recombination repair (HRR) is critical in BC. However, the prognostic role and immunotherapy response of HRR in BC remains to be clarified. Firstly, we identified HRR types in BC samples from the Cancer Genome Atlas (TCGA) and Gene Expression Omnibus (GEO) dataset (GSE42568) based on 65 HRR genes (HRRGs). A differentially expressed gene (DEG) list for different HRR types was generated. Then, the influences of gene sets composed of these DEGs on biological pathways and BC prognosis were explored. Next, we identified gene clusters based on gene sets composed of DEGs. Genes associated with prognosis for DEGs were identified using univariate Cox regression. Finally, the HRR score was constructed based on genes associated with prognosis. We analyzed how HRR score correlates with tumor mutation burden (TMB), immune cell infiltration (ICI), and immunotherapy response. Three HRR clusters were discovered. HRR subtype A demonstrated decreased infiltration and a high number of immunosuppressive cells with a poor prognosis. DEGs among various HRR types were predominantly enriched in cell cycle and genomic stability-related pathways. The prognostic model based on sixteen DEGs accurately predicted BC prognosis. The HRRGs were differentially expressed in three DEG clusters. TMB, ICI, and immunotherapy responses differed significantly between the high and low HRR groups (HSG, LSG). The HSG was distinguished by a high degree of ICI and low TMB. LSG had a better response to anti-PD-1 or anti-PD-1 and anti-CTLA4 combination therapy. This work revealed that HRR patterns would contribute to predicting prognosis and immunotherapy response in BC, which may benefit patients.

## Introduction

Breast cancer (BC) is the leading cause of incidence and mortality among women worldwide (Ahmad 2019). The number of new cases of female BC reported worldwide in 2018 reached approximately 2.1 million, with the incidence and mortality being 24.2% and 15%, respectively (Bray et al. 2018). Although there has been much progress in immunotherapy and targeted therapy for BC, the low overall response continues to be a hurdle, and the prognosis remains poor for patients with recurrent or metastatic BC (Ahmad 2019; Gerber et al. 2010; Varadé et al. 2021). Additionally, BC is a heterogeneous disease, so the prognosis for the same tumor node metastasis (TNM) stage and immunohistochemical subtype can be quite different (Yersal and Barutca 2014). Thus, exploring the cellular and molecular biological mechanisms of BC is essential for its treatment.

Previous studies have identified different BC subtypes according to immune cell infiltration status, DNA methylation, molecular apocrine, and basal-like-enrichment (Shen et al. 2020a; Jézéquel et al. 2019; Zhang et al. 2018), which confirmed the role of immunity and DNA mutations in BC. The tumor microenvironment (TME) was previously implicated in cancer progression and immunotherapy responses (Mittal et al. 2018; Schlam et al. 2021). The accumulation of mutations is a crucial part of cancer development, and repairing DNA damage is crucial to maintaining genomic integrity (Reilly et al. 2019). Breaks of DNA double-strand (DSB) in cells can hinder chromosomal replication, causing a chromosomal deletion. Cell death and tumor transformation will occur if it is not repaired promptly. Homologous recombination repair (HRR) is the most accurate and high-fidelity DNA damage repair approach responsible for DSB repair, involving many genes, including BRCA1 and BRCA2 (Neiger et al. 2021). ADP-ribose polymerase (PARP) is crucial for DNA single-strand break repair, mainly through base excision. Both BRCA1 and BRCA2 dysfunction may cause chromosome instability, cell-cycle arrest, and apoptosis. Dysfunction of either BRCA1 or BRCA2 seems to originate from PARP inhibition and can cause persistent DNA damage, which is usually repaired by HRR. PARP inhibitors (PARPi) selectively kill HRR deficiency tumor cells using the principle of synthetic lethality (Prados-Carvajal et al. 2021).

Our study integrated HRR-related genes (HRRGs) and identified HRR-related subtypes in BC based on public databases. After obtaining differentially expressed genes (DEGs) across HRR subtypes, a prognostic model was constructed based on them. Then, the biological functions of these DEGs were explored. We established the HRR score based on DEGs associated with BC prognosis and investigated the association between the HRR score and tumor mutation burden (TMB), immune cell infiltration (ICI), and therapeutic sensitivity. The research may provide an avenue for prognosis and immunotherapy response prediction in BC.

## Materials and methods

### Breast cancer data retrieval and homologous recombination repair-related genes

One thousand two hundred twelve BC samples were analyzed from two high-throughput platforms: 1091 from the TCGA and 121 from the GEO dataset (GSE42568). GEO database inclusion criteria are as follows. (1) “Breast cancer,” “Gene expression,” and “Homo sapiens” were used as the search keywords. (2) Entry type was set as “Series.” (3) Experiment type was “Expression profiling by array to facilitate subsequent analysis with R software.” (4) Samples involved in the study contain the gene expression profiles of breast cancer and normal breast biopsies. (5) Prognostic information of the patients was recorded. GEO database exclusion criteria are as follows. (1) The sample number is less than 50 cases. (2) RNA sequencing data and prognosis information of patients were incomplete. TCGA samples were downloaded with gene expression data, copy number variations (CNV), somatic mutation characteristics, clinical information, and survival data. GEO dataset samples were downloaded with annotated platform data and expression matrices. Probe IDs were converted to gene symbols using Perl. Patients with incomplete RNA-seq data and with insufficient survival data were excluded. TCGA gene expression data expressed as Fragments Per Kilobase Million (FPKMs) was transformed to Transcripts Per Million (TPMs). The “ComBat” function was used in the “SVA” package (version 3.50.0) to combine samples for further analysis (Leek et al. 2012). In total, 65 HRR-related genes (HRRGs) were acquired from the Molecular Signature (MsigDB, http://software.broadinstitute.org/gsea/msigdb) (Liberzon et al. 2015). Waterfall plots visualized somatic mutations with the “maftools” package (version 2.18.0) in R software inMayakonda et al. (2018), and HRRGs with CNV were visualized on 23 chromosomes with the “RCircos” package (version 1.2.2) (Zhang et al. 2013).

### Unsupervised clustering using homologous recombination repair genes

To identify various HRR-related patterns mediated by HRRGs, the expression of these 65 HRRGs was extracted from the integrated datasets. Finally, we obtained the expression data of 37 HRRGs, because genes from only one expression cohort were removed during the integration process (Stockwell et al. 2017; Hassannia et al. 2019; Bersuker et al. 2019; Doll et al. 2019; Zhu et al. 2021). Hierarchical agglomerative clustering was performed by the “ConsensusClusterPlus” package (version 1.66.0) (Wilkerson and Hayes 2010). An unsupervised analysis determined the number of clusters and subtypes. This process was repeated 1000 times to ensure a stable clustering.

### Gene set variation analysis

We conducted a gene set variation analysis (GSVA) with “GSVA” package (version 1.50.0) to examine the biological mechanisms across HRR subtypes (Hänzelmann et al. 2013). From MSigDB, gene sets from Gene Ontology (GO) and Kyoto Encyclopedia of Genes and Genomes (KEGG) were retrieved for GSVA. The pathways significantly associated with HRR subtypes were shown in heatmaps.

### Estimation of immune cell infiltration (ICI)

The ICI in each sample was assessed and quantified using single-sample gene-set enrichment analysis (ssGSEA) provided by the “GSVA” package (Hänzelmann et al. 2013). Charoentong’s study which collected information about immune cell marker gene expression was used to generate an enrichment score. Then, the score was used to reflect each immune cell’s relative abundance of infiltration. The ICI variations among HRR subtypes were subsequently examined.

### Identification of differentially expressed genes among homologous recombination repair subtypes in breast cancer

Unsupervised clustering results were used to separate BC samples into different subtypes based on HRR expression patterns. We screened differentially expressed genes (DEGs) across subtypes using “Limma” package (version 3.46.0) in R software (Ritchie et al. 2015). An adjusted false discovery rate (FDR) of 0.05 was used as a threshold, as well as the absolute value of LogFC was greater than 1. The common DEGs up-regulated or downregulated of distinct subtypes were found via drawing Venn diagrams. The common DEGs from different HRR subtypes were submitted for GO and KEGG analysis to investigate their molecular biology using “clusterProfiler” package (version 4.10.0) (Yu et al. 2012). A *P*-value less than 0.05 was considered a statistically significant difference.

### Construction and validation of prognostic model of breast cancer

We used the Cox regression model to investigate the influence of multiple factors on survival. The integrated dataset was divided into training and validation sets at random. The model was built on the training set and then evaluated on the validation. To select genes associated with survival, we used *P* = 0.05 as a filter for univariate Cox. “glmnet” package (version 4.1–8) was used for LASSO regression analysis and determining the K value through minimal lambda to avoid significant variances (Engebretsen and Bohlin 2019). Subsequently, risk genes were identified using multivariate Cox regression, and a prognostic risk model was developed. Gene expression and Cox regression coefficients were used to calculate the risk score. Patients were grouped into high-risk and low-risk groups based on their median risk scores in training, validation, and total sets. To determine whether high-risk and low-risk groups had similar survival rates, we used Kaplan–Meier (K-M) analysis, to draw survival curves (Wu et al. 2021). The time-dependent receiver operating curves (ROC) were drawn using the R package “Survival ROC” (version 1.0.3.1) to evaluate the model’s predictive ability and stability over 1-year, 3-year, and 5-year periods (Liu et al. 2021a). The area under the curve (AUC) > 0.5 and closer to 1 indicates a better prognosis. Additionally, clinical information (age, sex, and stage) was unitized in this prognostic risk model for clinical application.

### Construction of homologous recombination repair score

The HRR score was developed to measure the features of HRR in BC. Genes with significant prognosis differences were found through the above methods. Unsupervised clustering was used to group patients for an in-depth examination. Then, these DEGs’ primary components were extracted using principal component analysis (PCA), and the HRRG signatures were created. Signature scores were chosen from principal components 1 and 2 (Ringnér 2008). In order to calculate the HRR score of each patient, the following equation was used: HRR score = ∑PCA1*i* + ∑PCA2*i* (*i* is the expression of prognostic DEGs). With the “maxstat” package (version 0.7–25), we obtained the best cutoff value for separating High and Low HRR score groups (HSG and LSG) linked to prognosis (Ogłuszka et al. 2019).

### Prediction of immunotherapy sensitivities

To compare HSG and LSG expression differences, we downloaded the immunophenoscore (IPS) data from the Cancer Immune Atlas (TCIA) database (http://tcia.at/) (Guo et al. 2022). Then, differences in anti-cytotoxic T-lymphocyte antigen 4 (CTLA-4) and anti-programmed cell death protein 1 (PD-1) antibody responses were compared between different HRR score groups to predict immunotherapy sensitivity.

### Statistical analysis

Statistical analysis in this study was performed using R software (version 4.0.5). A comparison of numerical type variables between two or more groups was conducted using Wilcoxon and Kruskal–Wallis tests. Kaplan–Meier curves were used for drawing prognosis and survival curves, and the chi-square test was used for categorized variable comparisons between HSG and LSG groups. The correlation coefficient was calculated using Spearman’s test. A *P-*value < 0.05 was considered a statistically significant difference.

## Result

### Genetic variations in homologous recombination repair genes in breast cancer

**Fig. 1:**
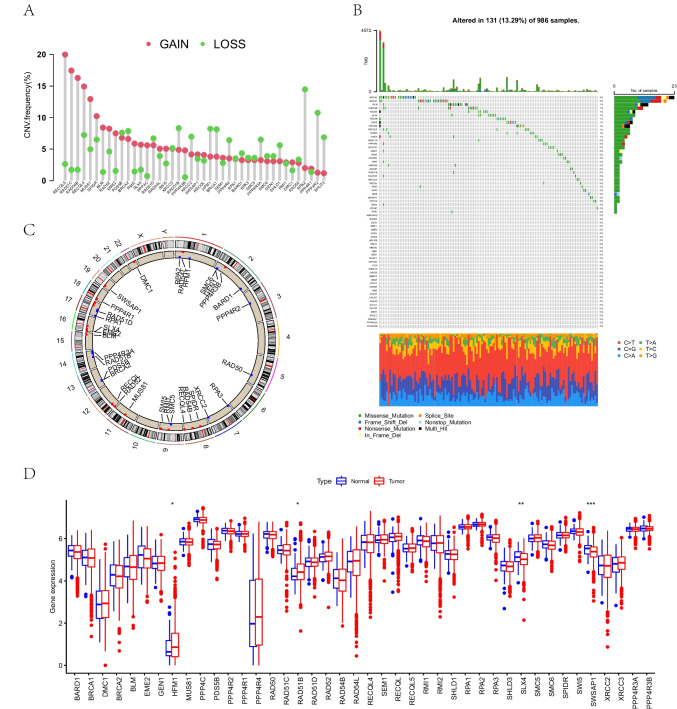
The CNV frequency of HRRGs and expression differences of HRRGs in normal and tumor tissues in the TCGA-BRCA cohort. **A** The CNV frequency of HRRGs in the TCGA cohort. The points represent the CNV frequency of HRRGs in the TCGA cohort. The height of the column indicates the frequency of CNV change. The green dots indicate the frequency of deletions, and the red ones indicate the amplification frequency. **B** Frequency of HRRG mutations in 1090 patients from the TCGA-BRCA cohort. The columns represent patients, and the different colors at the bottom of the figure represent the proportion of different types of mutations. **C** The position of HRRGs with CNV on 23 chromosomes. **D** The expression differences of HRRGs in normal tissues and tumor tissues. CNV, copy number variation; HRRGs, homologous recombination repair genes; BRCA, breast cancer

There were 65 HRRGs in this study. First, we estimated and displayed that HRRGs had frequent CNV changes in BC and that most HRRGs were concentrated on CNV amplifications, while some HRRGs had a higher frequency of CNV deletions (Fig. 1A). RECQL5, RAD51C, and RAD54B were the genes with the highest copy number amplification frequency. RPA2, PPP4R2, and RAD51B were among the genes with the highest prevalence of deletions. HRRG mutations were found in 131 of 986 samples, suggesting a 13.29% mutation frequency. Among 42 HRRGs carrying mutations, a 2% mutation frequency was found in BRCA2, followed by BRCA1 (Fig. 1B). The findings revealed that CNV was expected, and deletions or amplifications of different HRRGs had distinct features. The location of the mutated genes on 23 pairs of chromosomes and the prevalence of CNVs on HRRGs are shown in Fig. 1C. HRRG expression levels differed between tumor and normal samples, as shown in Fig. 1D. Some HRRGs, such as HFM1 and PPP4R4, were higher expressed significantly in tumors, while some genes, such as EME2 and MUS81, were lower expressed in tumors, which reminded us that BC may be triggered and progressed by differences in HRRG expression.

### Identification of homologous recombination repair phenotypes based on gene expression patterns

**Fig. 2:**
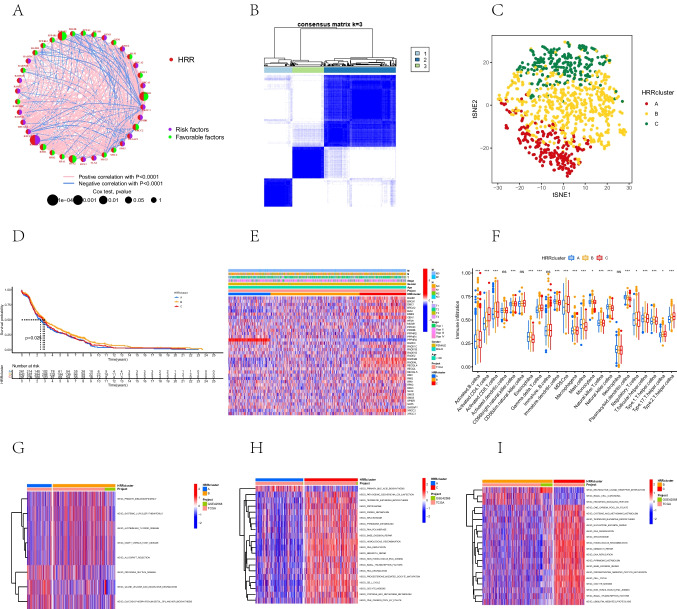
Identification of HRR clusters and ICI features and biological characteristics in different HRR clusters. **A** Interaction between HRRGs. The size of the circle indicates the range of prognostic significance of each HRRG. *P*-values were calculated by the log-rank test. Blue dots represent protective factors for prognostic. Red dots represent risk factors for prognosis. The connected lines represent their correlation. The thickness of line indicates the correlation strength. Gray and yellow lines show negative and positive correlations, respectively. **B** The consensus matrix of all samples. All samples were clustered into the appropriate number of subtypes (*k* = 3). **C** The distribution of the three HRR subtypes is shown by tSNE dimension reduction. **D** Kaplan–Meier curves for the difference in overall survival between HRR subtypes A, B, and C (*P* = 0.025). **E** The heatmap of the expression of HRRGs in different HRR clusters. The color of the heatmap indicates the relative expression of HRRGs. **F** Abundance of each ICI in HRRG clusters A, B, and C. **G** The heatmap of top 20 biological pathways in HRR clusters A and B through GSVA. Red and blue represent activated and inhibited pathways, respectively. **H** The heatmap of top 20 biological pathways in HRR clusters A and C through GSVA. **I** The heatmap of the top 20 biological pathways in HRR clusters B and C through GSVA. HRRGs, homologous recombination repair genes; ICI, immune cell infiltration; GSVA, gene set variation analysis

The interaction relationships and prognostic effects of HRRGs were further explored with multivariate COX and correlation analyses. Thirty-seven HRRGs had effects on the prognosis of BC patients, with some HRRGs having a significant positive correlation with prognoses, such as SLX4, RAD54L, RECQL4, and RECQ, and some HRRGs, including RAD51B, RAD50, and PD55B, were negatively correlated with prognosis (Fig. 2A). Additionally, the favorable prognostic HRRGs were negatively correlated with the unfavorable HRRGs. For example, there was a negative association between SLX4 and RAD51B, RAD50, and PD55B. The complex interplay between the HRRGs may play a crucial role in patient prognosis and ICI characteristics. Subsequently, we performed a cluster analysis based on differential expression patterns of HRRGs using the R package “ConsensusClusterPlus” (version 1.66.0). Three HRR-related phenotypes were identified and labeled as HRR clusters A, B, and C (Fig. 2B). The clustering effect was good in three groups (A, B, and C) according to T-SNE analysis (Fig. 2C). A cluster with a worse prognosis than clusters B and C was revealed by the KM curves (*P* = 0.025) (Fig. 2D). Heatmaps showed HRRG expression patterns, and we can conclude that HRRGs were expressed differently in each cluster (Fig. 2E).

### Immune cell infiltration features and biological characteristics in different homologous recombination repair phenotypes

Activated dendritic cells, CD56 bright natural killer (NK) cells, neutrophils, and immature B cells did not differ significantly, as ICI analysis showed. Most of the immune cells in the subtype A cluster had lower infiltration. It was flooded with immune-suppressive cells, including eosinophils, myeloid-derived suppressor cells (MDSCs), macrophages, mast cells, and Tregs, which may be related to different prognoses (Fig. 2F). Thus, we speculated that HRR might cause immunosuppression and accelerate tumor progression by suppressing immunity.

The biological behaviors among three HRR-related subtypes were explored through GSVA analysis (Fig. 2G–I). In cluster A, valine, leucine, and soleucine degradations; primary immunodeficiency; and other related signals were significantly involved. Signals, including DNA replication, mismatch repair, and homologous recombination, were mainly enriched in cluster C. On the contrary, cluster B was primarily enriched in complement, coagulation cascades, and ECM receptor interactions.

### Screening of homologous recombination repair phenotype-related differentially expressed genes and functional enrichment analysis

**Fig. 3:**
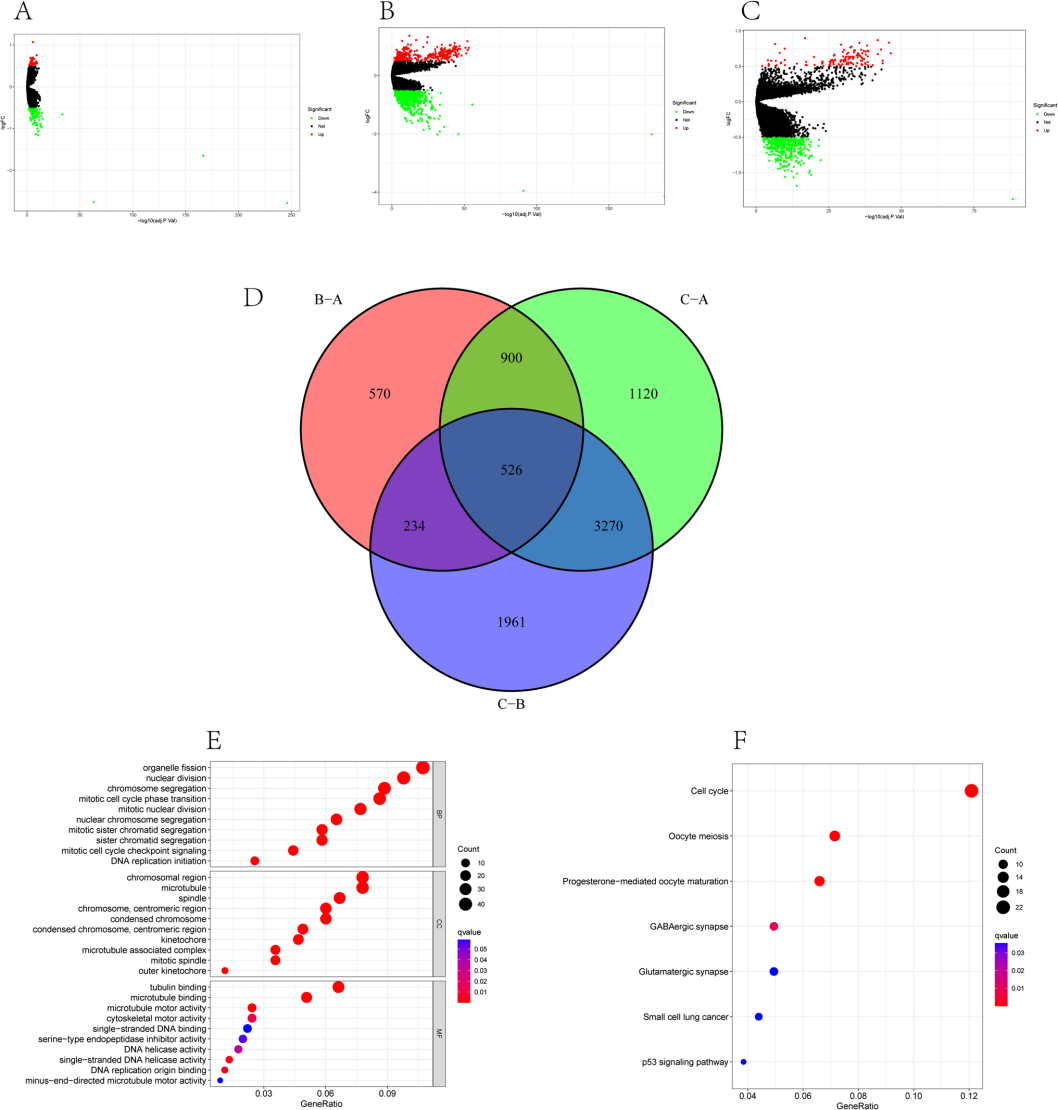
Screening of DEGs among HRR clusters and functional enrichment analysis of DEGs. **A** Volcano map of group A and B DEGs. **B** Volcano map of group A–C DEGs. **C** Volcano map of group B and C DEGs; **D** Venn diagram of DEGs in three groups (A, B, and C). **E** GO enrichment analysis of DEGs. **F** KEGG enrichment analysis of DEGs. DEGs, differentially expressed genes; GO, Gene Ontology; KEGG, Kyoto Encyclopedia of Genes and Genomes

DEGs among these three subtypes have been determined, and the results were visualized using volcano plots (Fig. 3A–C). There were 2230 DEGs between subtypes A and B, 5991 DEGs between subtypes B and C, and 5816 DEGs between subtypes A and C. Five hundred twenty-six common DEGs were identified by intersecting the DEGs of the three groups (Fig. 3D). We used these DEGs for functional enrichment analysis. The critical GO biological processes were organelle fission, chromosomal region, and tubulin binding (Fig. 3E). Analysis of KEGG showed that the genes were primarily involved in the cell cycle, oocyte meiosis, and progesterone-mediated maturation of oocytes (Fig. 3F).

### Construction and validation of the prognostic risk model

**Fig. 4:**
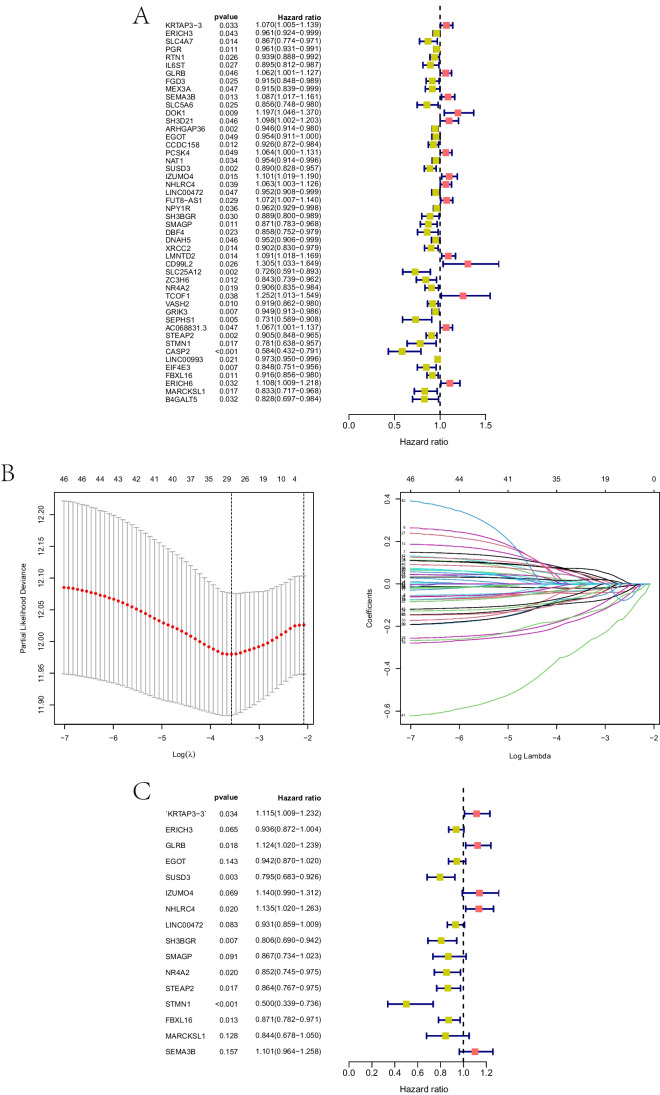
Results of univariate Cox regression, LASSO regression, and multivariate Cox regression analysis. **A** Forest plot of univariate Cox regression analysis. **B** LASSO regression for univariate results. **C** Forest plot of multivariate Cox regression analysis

**Fig. 5:**
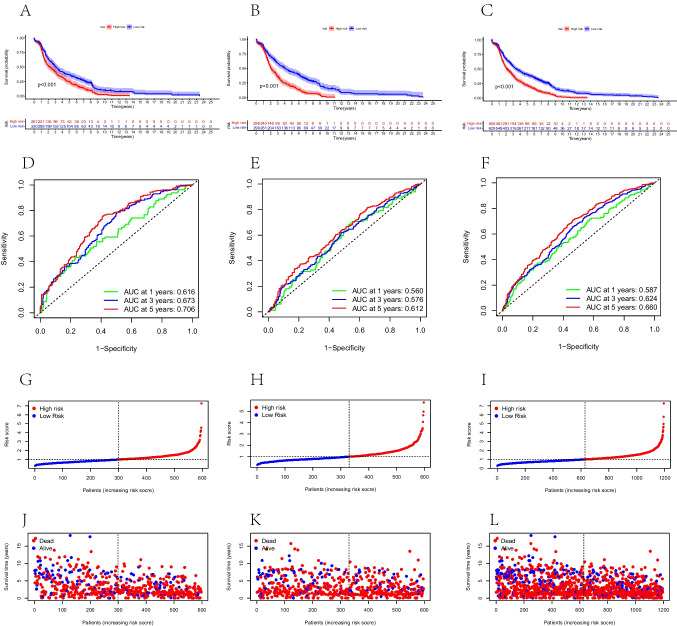
Kaplan–Meier survival curves, time-dependent ROC curves, and survival status maps of the total data set, training set, and validation set. **A**–**C** Kaplan–Meier survival curves and risk curves of the total data set, training set, and validation set. **D**–**F** Time-dependent ROC curves of the total data set, training set, and validation set. **G**–**L** Survival status maps of the total data set, training set, and validation set. ROC, receiver operating characteristic

The prognostic model was constructed using 1194 samples with complete gene expression and survival data. Samples were split into training and validation sets randomly. Forty-eight DEGs were related to prognostic survival in the univariate Cox regression (*P* < 0.05) (Fig. 4A). Lasso regression was performed to prevent overfitting, and 28 genes related to prognosis were obtained (Fig. 4B). A risk model was constructed using 16 DEGs through multivariate COX analysis, and a risk score was calculated to assess the prognosis (Fig. 4C). The 16 genes included were KRTAP3-3, ERICH3, GLRB, EGOT, SUSD3, IZUMO4, NHLRC4, LINC00472, SH3BGR, SMAGP, NR4A2, STEAP2, STMN1, FBXL16, MARCKSL1, and SEMA3B. Calculations of risk scores were performed as follows: Risk score = (0.108 × KRTAP3-3) + (− 0.06 × ERICH3) + (0.117 × GLRB) + (0.05 × EGOT) + (− 0.229 × SUSD) + (0.13 × IZUMO4) + (0.126 × NHLRC4) + (0.07 × LINC00472) + (0.215 × SH3BGR) + (− 0.143 × SMAGP) + (− 0.16 × NR4A2) + (− 0.145 × STEAP2) + (− 0.693 × STMN1) + (− 0.137 × FBXL16) + (− 0.169 × MARCKSL1) + (0.096 × SEMA3B). The median risk scores of BC patients in total, training, and validation sets were used to categorize them into high- and low-risk groups. There was a worse survival rate among all three sets of patients who had high-risk scores (*P* < 0.001) (Fig. 5A–C). In the total set, AUC at 1 year was 0.616; at 3 years, 0.673; and at 5 years, 0.706 (Fig. 5D). The AUC for the training set was 0.560 after 1 year, 0.576 after 3 years, and 0.612 after 5 years (Fig. 5E). Additionally, the AUC was 0.587 after 1 year, 0.624 after 3 years, and 0.660 after 5 years in the validation set (Fig. 5F). This model predicted BC prognosis well, with good sensitivity and specificity. The visualized risk scores showed increased mortality and decreased survival time, along with the increase in risk scores in the total set, training set, and validation set, respectively (Fig. 5G–L).

### Identification of prognostic-related subtypes based on secondary clustering using differentially expressed genes

**Fig. 6:**
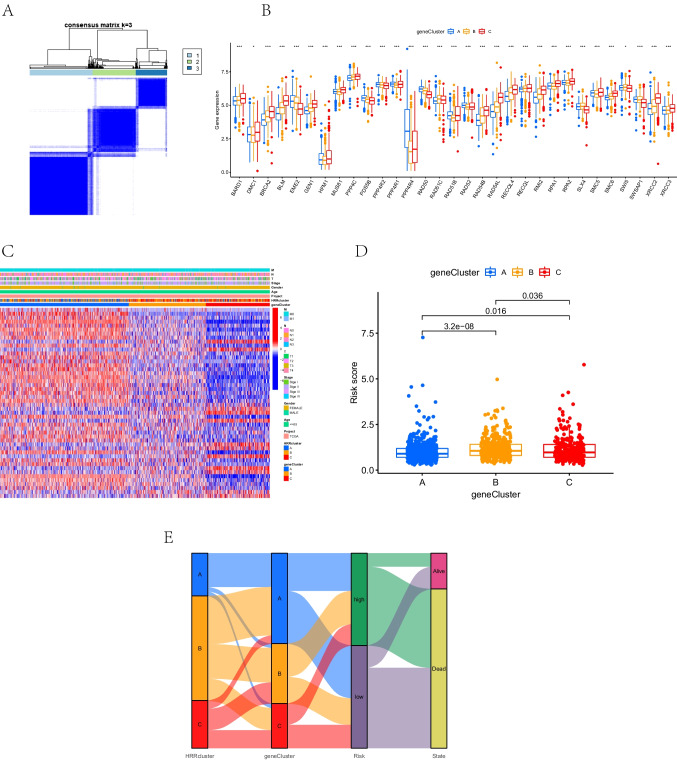
Construction of gene clusters based on HRR cluster-related DEGs.** A** Consensus matrix based on the TCGA-BRCA cohort in three HRR clusters. TCGA samples were clustered into the appropriate number of subtypes (*k* = 3). **B** The differential expression of HRRGs in different gene clusters. **C** Heatmap of the HRR-related DEGs in different HRR clusters and gene clusters. **D** Differences in HRR scores among the three gene clusters. The statistical differences among the three gene clusters were compared by the Wilcoxon test (*P* < 0.05). **E** Sankey diagram of the distribution of patients with HRR cluster, gene cluster, risk group, and survival status. Positive correlation is marked in red and negative correlation in blue. HRR, homologous recombination repair; DEGs, differentially expressed genes; BRCA, breast cancer

To determine the biological characteristics of HRR subtypes, we conducted an unsupervised cluster analysis of 526 overlapping DEGs. We identified three DEG clusters (gene clusters A–C) associated with the HRR phenotype (Fig. 6A). There were significant differences in the expressions of HRRG gene clusters (Fig. 6B). The heatmap illustrated HRRG expression patterns and showed differential expression of HRRGs in three gene clusters (Fig. 6C). There was a lower risk score of cluster A than that of clusters B and C (Fig. 6D). The Sankey diagram visualized each patient’s HRR clusters, gene clusters, risk group, and survival status (Fig. 6E). High-risk patients were most likely to have poor prognoses in gene cluster A. In gene cluster B, the majority of patients having poor prognoses were at high risk.

### Correlation between homologous recombination repair score and tumor mutation burden

**Fig. 7:**
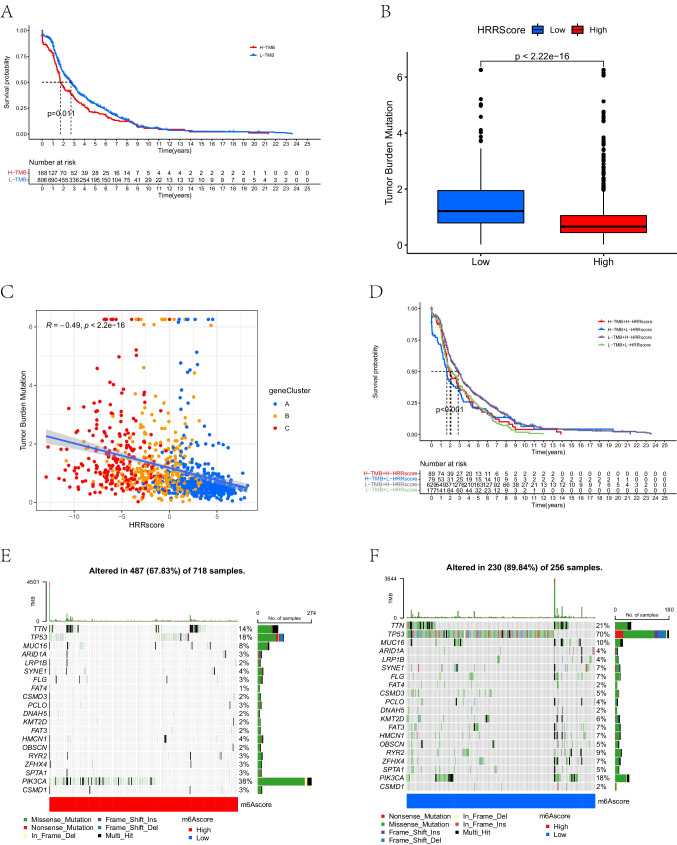
The impact of TMB on prognosis and its association with HRR score. **A** Kaplan–Meier curves of the overall survival difference between high- and low-TMB groups (*P* = 0.011). The statistical differences were compared by the Wilcoxon test. **B** The TMB levels between the HSG and LSG groups. **C** Correlation between HRR score and TMB (Spearman coefficient). **D** Kaplan–Meier curves for the differences in overall survival stratified by TMB and HRR score (*P* < 0.001). **E**, **F** Waterfall plots for top 20 driver genes with the highest mutation frequency in HSG (**E**) and LSG (**F**). TMB, tumor mutation burden; HRR, homologous recombination repair; HSG, high HRR score group; LSG, low HRR score group

A series of somatic mutation analyses were made on some genome stability pathways involved in the GSVA analysis. The impact of TMB on prognosis was explored. As shown in Fig. 7A, the higher TMB group had a worse prognosis. As shown in Fig. 7B, compared to LSG, HSG had a lower TMB level (*P* < 0.001). TMB and HRR scores correlated negatively (Spearman coefficient: *r* = 0.49, *P* < 0.001, Fig. 7C). We performed a stratified prognostic analysis based on the synergism of the TMB and HRR scores. High HRR and low TMB scores were associated with a significant survival advantage for patients (Fig. 7D), which revealed that the HRR score could indicate prognosis effectively. After this, we analyzed the differences between HSG and LSG in terms of the distribution of somatic mutations. The frequency of mutations of the top 20 genes with the most mutations was generally lower in HSG than in LSG (Fig. 7E, F).

### Correlation between HRR score and immune cell infiltration

**Fig. 8:**
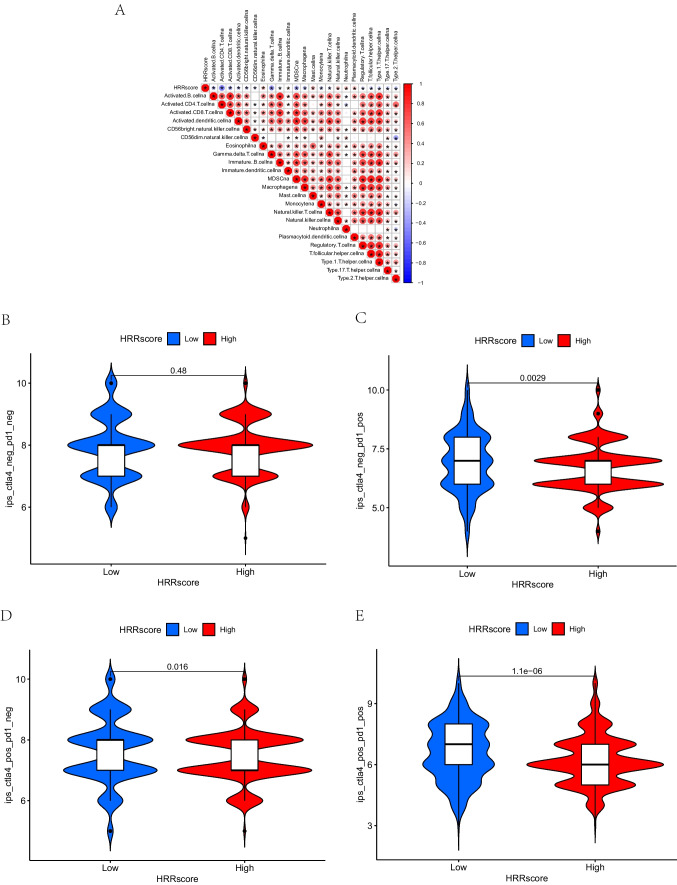
The association between HRR score and ICI and differences of IPS between HSG and LSG. **A** Spearman analysis of the correlation between HRR score and ICI abundance in the TCGA cohort. **B** In PD-1_NEG + CTLA4_neg, there was no significant difference between the IPS of the HSG and the LSG. **C**–**E** In PD-1_NEg + CTLA4_pos, CTLA4_pos + PD-1_POS, and PD-1_POS + CTLA4_neg, IPS increased in the LSG. HRR, homologous recombination repair; ICI, immune cell infiltration; IPS, immunophenoscores; HSG, high HRR score group; LSG, low HRR score group

Based on DEG expression patterns, we developed an algorithm using PCA and defined the result as an HRR score. We determined the optimal cutoff value using the “maxstat” package. Due to the differences in ICI characteristics among HRR subtypes, we also examined the association between HRR score and ICI (Fig. 8A). A positive correlation was demonstrated between HRR scores and most infiltrating immune cells, following the results of HRR subtypes.

### Correlation between HRR score and immunotherapy response

A comparison of the IPS difference between HSG and LSG in four types (CTLA4_negative + PD-1_negative, CTLA4_positive + PD-1_negative, CTLA4_negative + PD-1_positive, CTLA4_positive + PD-1_positive) was conducted in order to determine if HRR score would predict the clinical immunotherapy effects of BC, visualized by an immunophenotype map (Fig. 8 B–E). The result indicated that for PD-1_NEG + CTLA4_neg types, no significant difference existed between the IPS of the HSG and the LSG (Fig. 8B). However, for PD-1_NEg + CTLA4_pos, CTLA4_pos + PD-1_POS, and PD-1_POS + CTLA4_neg types, IPS increased in the LSG (Fig. 8C–E). Results showed that patients in the LSG responded better to anti-PD-1 therapy or anti-PD-1 and anti-CTLA4 therapy.

## Discussion

In this study, we identified three HRR clusters based on HRRGs. The three HRR clusters showed different ICI characteristics and HRRG expression patterns. DEGs identified in HRR clusters were primarily involved in pathways related to cell cycle and genome stability. The prognostic model based on 16 DEGs accurately predicted BC prognosis. We also identified three gene clusters based on DEGs, which could identify prognostic subtypes more stably. High ICI, low TMB, and worse immunotherapy response were observed in HSG. The results may help predict prognosis and potential therapeutic benefits.

We mapped three clusters of HRR-related DEGs and built a prognostic nomogram for BC. The ROC curves showed that this model was good at predicting BC prognosis. The nomogram contained 16 DEGs. The majority of these genes play a role in the development of BC and other types of cancers. Researchers have found that ERICH1-AS1 expression can predict non-small cell lung cancer prognosis (Tang et al. 2015). BC cells can be triggered to proliferate and migrate by SUSD3 (Moy et al. 2015). EGOT has also been used to construct a risk prediction model related to BC (Lv et al. 2021). By inactivating the Hedgehog signaling pathway in BC, overexpression of EGOT reduces the viability and migration of the cells (Qiu et al. 2020). Cells can become more sensitive to paclitaxel toxicity by increasing the levels of EGOT, and regulating EGOT may be a new way to boost paclitaxel toxicity (Xu et al. 2019). Up-regulation of LINC00472 inhibited the viability, invasion, migration, and EMT of lung cancer cells but increased the apoptosis rate of lung cancer cells (Mao et al. 2019). Cell growth in vitro and xenograft tumor growth in vivo in BC tumors were inhibited where SH3BGRL2 was downregulated (Li et al. 2020). SMAGP knock-down can inactivate the PI3K/Akt pathway, thereby inhibiting the malignant phenotypes of glioblastoma cells (Ni et al. 2020). Overall survival and recurrence-free survival were positively associated with NR4A1-NR4A3 expression. Additionally, NR4A family genes regulate oxidative phosphorylation in BC (Yousefi et al. 2022). Downregulation of STEAP2 expression is associated with a poor prognosis for BC. STEAP2 acts as an anti-oncogene during BC development. Through PI3K/AKT/mTOR signaling, STEAP2 downregulation can promote tumor cell proliferation and metastasis (Yang et al. 2020). MiR-770 inhibits BC metastasis by targeting STMN1 directly (Li et al. 2018). The high level of STMN1 protein in BC tissues was related to poor prognosis (Tang et al. 2020). Angiogenesis and epithelial-mesenchymal transition in BC can be blocked by FBXL16. BC with FBXL16 downregulation has a higher node and high-grade tumors and poor survival (Kim et al. 2021). In lung adenocarcinoma cells, MARCKSL1 promotes proliferation, migration, and invasion (Liang et al. 2020). SEMA3B-AS1 could target the miR-3940/KLLN axis to inhibit BC progression (Hu et al. 2022). This proved that the 16 DEGs in nomograms were mostly able to play a role in the development and prognosis of BC and other types of cancers.

In HRR cluster A, most immune cells had lower infiltration, including immunosuppressive cells, such as eosinophils, MDSCs, macrophages, mast cells, and Tregs. It has been shown in previous studies that patients with a low eosinophil count have a higher recurrence rate and a worse prognosis (Ownby et al. 1983; Onesti et al. 2020). Innate and adaptive immunity are inhibited by MDSCs, which are heterogeneous groups of immature myeloid cells. MDSC expansion triggers the pre-cancerous immune microenvironment, thus accelerating BC progression (Liu et al. 2021b). The mononuclear phagocytic system includes macrophages. They are usually broadly classified into M1 and M2. M1 macrophages might help metastatic BC cell dormancy, while M2 macrophages might promote tumor outgrowth (Lin et al. 2019). The increased mast cell density and distribution are a worse prognostic factor for BC (Carpenco et al. 2019). Treg cells are known to play a crucial role in peripheral tolerance and can suppress effector T cells to prevent unwanted immune responses. Treg cells have an invasive appearance and are associated with a reduced chance of relapse-free survival and overall survival in BC biopsies (Mittal et al. 2018; Togashi et al. 2019). A decrease in Tregs and naive CD4 + T cells in TME might suppress BC metastasis (Li et al. 2021). These results suggested that HRR subtypes were associated with ICI in TME. So, we analyzed how the HRR score correlated with ICI and found a positive correlation between HRR scores and most infiltrating immune cells.

A positive link between HRR score and immune cells infiltrating indicated the possible association between HRR and immunity. The HRRGs had frequent CNV amplifications and deletions, especially in LSG. HRR-related DEGs tended to be enriched in cell cycle and genome stability. HRR is the most accurate and high-fidelity way to repair DNA damage. Inherited mutations in HRRGs can increase the risk of BC development (Mersch et al. 2015). TMB elevation is caused by mutations in the DNA damage response genes, predicting a poor prognosis (Klempner et al. 2020). We found that TMB was negatively related to survival and HRR scores. Combining TMB and HRR scores can further improve prognosis prediction. It has been reported that immunotherapy might benefit patients with poor prognoses because their TMB is higher and their genes are more mutated (Barroso-Sousa et al. 2020; Liu et al. 2020). In line with previous research, anti-PD-1 therapy or anti-PD-1 and anti-CTLA4 therapy worked better for patients in the LSG. Significant breakthroughs have been made in some advanced therapies, including PARP-targeted therapy. There are several PARPi approved to treat BC, including taraparib and olaparib (Lyons 2019; Shen et al. 2020b). PARPi can further hinder DNA repair in tumor cells in patients with HRR deficiency, accelerating their death and achieving precise targeting (Chopra et al. 2020).

In this study, we identified three HRR subtypes and comprehensively explored the association between HRR and ICI. Our gene signature model predicts BC prognosis with high accuracy based on HRR-related DEGs, which may help develop new treatment options. The HRR score could predict TMB and immunotherapy responses, which may provide references for clinical treatment.

## Data Availability

The data are available at the GEO database (https://www.ncbi.nlm.nih.gov/geo/) (GSE42568), TCGA database (http://cancergenome.nih.gov/), and TCIA database (https://tcia.at/).
